# Cauliflower mosaic virus disease spectrum uncovers novel susceptibility factor *NCED9* in *Arabidopsis thaliana*

**DOI:** 10.1093/jxb/erad204

**Published:** 2023-05-30

**Authors:** Gesa Hoffmann, Aayushi Shukla, Silvia López-González, Anders Hafrén

**Affiliations:** Department of Plant Biology, Uppsala BioCenter, Swedish University of Agricultural Sciences, 75007 Uppsala, Sweden; Linnean Center for Plant Biology, 75007 Uppsala, Sweden; Department of Plant Biology, Uppsala BioCenter, Swedish University of Agricultural Sciences, 75007 Uppsala, Sweden; Linnean Center for Plant Biology, 75007 Uppsala, Sweden; Department of Plant Biology, Uppsala BioCenter, Swedish University of Agricultural Sciences, 75007 Uppsala, Sweden; Linnean Center for Plant Biology, 75007 Uppsala, Sweden; Department of Plant Biology, Uppsala BioCenter, Swedish University of Agricultural Sciences, 75007 Uppsala, Sweden; Linnean Center for Plant Biology, 75007 Uppsala, Sweden; Max Planck Institute for Molecular Plant Physiology, Germany

**Keywords:** Abscisic acid, Arabidopsis, cauliflower mosaic virus, genome-wide association studies, virus disease, virus tolerance

## Abstract

Viruses are intimately linked with their hosts and especially dependent on gene-for-gene interactions to establish successful infections. On the host side, defence mechanisms such as tolerance and resistance can occur within the same species, leading to differing virus accumulation in relation to symptomology and plant fitness. The identification of novel resistance genes against viruses and susceptibility factors is an important part of understanding viral patho­genesis and securing food production. The model plant *Arabidopsis thaliana* displays a wide symptom spectrum in response to RNA virus infections, and unbiased genome-wide association studies have proven a powerful tool to identify novel disease-genes. In this study we infected natural accessions of *A. thaliana* with the pararetrovirus cauliflower mosaic virus (CaMV) to study the phenotypic variations between accessions and their correlation with virus accumulation. Through genome-wide association mapping of viral accumulation differences, we identified several susceptibility factors for CaMV, the strongest of which was the abscisic acid synthesis gene *NCED9*. Further experiments confirmed the importance of abscisic acid homeostasis and its disruption for CaMV disease.

## Teaser

Natural variation in the *Arabidopsis thaliana*/cauliflower mosaic virus pathosystem uncovers a broad spectrum of disease outcomes and identifies the ABA biosynthesis gene *NCED9*as a novel susceptibility factor.

## Introduction

Plant viruses are ubiquitous in wild and cultivated habitats, with profound impacts on host populations (Prendeville *et al.*, 2012). As obligate intracellular parasites, they are fully dependent on host compatibility to complete their replication cycle, and genetic variation within both the plant and the viral species can have major effects on the disease outcome (Cecchini *et al.*, 1998; Butković *et al*., 2022, Preprint). Of particular interest is the continuum of two mechanisms, tolerance and resistance, that plants employ against invading pathogens. Host resistance leads to reduced or absent viral replication and commonly functions through targeted degradation of viral components and incompatibility with the host machinery (Soosaar *et al.*, 2005). Tolerance is fundamentally different from resistance and is defined as a mitigation strategy aimed at minimizing the cost of infection in terms of plant growth, yield and reproduction, rather than investing in resources to fight the infection by suppressing pathogen multiplication (Pagán and García-Arenal, 2020). The definition of tolerance can vary, and in the current study we refer to it when virus accumulation is high in visibly healthy plants. A powerful example was recently reported for Arabidopsis latent virus 1, which has spread through natural and laboratory populations of *Arabidopsis thaliana* without detection (Verhoeven *et al*., 2023). While agricultural research has historically focused on resistance to combat virus disease, evidence is accumulating that tolerance plays a pivotal role for many plant–virus interactions, especially in natural ecosystems, where most plants are infected by at least one virus at any given time but still appear healthy (Roossinck, 2013; Paudel and Sanfaçon, 2018). Identifying the underlying genetics of the tolerance–resistance spectrum is a difficult task, and genome-wide association studies (GWAS) have emerged as a potential tool to find novel genes and pathways implicated in plant–pathogen interactions (reviewed in Bartoli and Roux, 2017). Compared with other pathogen classes, GWAS on plant–virus interactions are scarce and most have focused on crop and vegetable species (reviewed in Monnot *et al.*, 2021) even though, thanks to the extensive 1001 Genomes project, Arabidopsis is a superb resource for GWAS, with over 1000 sequenced naturally inbred accessions collected worldwide (1001 Genomes Consortium, 2016). To our knowledge, six recent GWAS have been conducted on RNA virus infections in Arabidopsis (Pagny *et al.*, 2012; Rubio *et al.*, 2019; Butković *et al.*, 2021; Montes *et al.*, 2021; Butković *et al*., 2022, Preprint; Liu *et al.*, 2022) and successfully identified genetic loci impacting viral infections.

In addition to discovering new disease and resistance genes for possible application in crop breeding and protection strategies, natural genetic variation and associated phenotypic variation in virus accumulation and symptomology can suggest fundamental perspectives on plant–virus interactions. Only one of the six virus/Arabidopsis GWAS determined both symptomology and virus accumulation and found a weak positive correlation between the traits (Rubio *et al.*, 2019). Yet, plant viruses generally do not show a correlation between symptomology and accumulation across Arabidopsis accessions, as observed (Cecchini *et al.*, 1998; Pagán *et al.*, 2007; Shukla *et al.*, 2018; Bergès *et al.*, 2021), suggesting that tolerance is a ubiquitous process in plant viral diseases.

In this study we examined the disease spectrum of the double-stranded DNA Caulimovirus *Cauliflower mosaic virus* (CaMV; family *Caulimoviridae*) in 100 natural accessions of Arabidopsis. We focused our analysis on only the plant’s vegetative stage and its rosette tissue, owing to extensive vernalization requirements for many of the accessions to flower. The CaMV host range is limited to members of the *Brassicaceae*, including mustard, broccoli, and cabbage, and it infects natural populations of Arabidopsis (Pagán *et al.*, 2010). CaMV challenges its host with the establishment of large cytoplasmic viral replication centers, as well as an uncommon increase of global translation, due to the viral translational transactivator protein P6 (Schoelz and Leisner, 2017; Hoffmann *et al.*, 2022). The unique properties of CaMV implicate the existence of a network of host factors possibly influencing CaMV disease. Interestingly, CaMV infection was shown to cause a range of disease severity in response to water deficit in natural accessions of Arabidopsis (Bergès *et al.*, 2020), altogether making CaMV a suitable virus for a GWAS in Arabidopsis.

Here, we show that CaMV disease differs greatly among Arabidopsis accessions, dependent on the host genotype, and use this variety to map underlying host genes. We find that the abscisic acid (ABA) synthesis gene 9-*cis*-epoxycarotenoid dioxygenase 9 (*NCED9*) is an important susceptibility factor for CaMV, as infection is almost completely abolished in the *nced9* mutant line. Additionally, ABA, an important plant hormone in plant abiotic and biotic stress responses (Ton *et al.*, 2009; Verma *et al.*, 2016), is targeted during CaMV infection, and misregulation of ABA homeostasis increases CaMV levels.

## Materials and methods

### Plant material and growth conditions

*Arabidopsis thaliana* accessions (*n*=100) (Supplementary Table S1) were provided by the group of Magnus Nordborg (Gregor Mendel Institute, Vienna). The T-DNA lines used in this study were ordered from the Nottingham Arabidopsis Stock Centre (NASC) and all generated in the Columbia (Col-0) background, which was used as a control for all mutant experiments (Supplementary Table S2). Seeds were planted on damp soil and stored at 4 °C in the dark for 1 week to ensure germination synchronization. Seedlings were separated into pots at six plants per pot 8 d after transfer to a walk-in chamber in short-day conditions (120 mmol, 10 h light/14 h dark cycle) at 22 °C and 65% relative humidity. Pots were randomized within each tray and tray position within the chamber was switched randomly once a week. Infections were carried out 18 d after transfer to growth conditions. Infections of natural accessions were repeated twice in timely separated experiments. T-DNA lines were infected at least three times in timely separated experiments. Arabidopsis plants were grown in walk-in chambers in standard long-day conditions (16 h light/8 h dark cycle) at 22 °C and 65% relative humidity for propagation. For long day infection experiments, seeds were plated on damp soil and stored at 4 °C in the dark for 1 week to ensure germination synchronization. Seedlings were separated into four plants per pot 6 d after transfer to a walk-in chamber and infections were carried out 15 d after transfer to growth conditions.

### Virus inoculation and symptom scoring

All Arabidopsis plants were infected with CaMV at growth stage 1.04 with four rosette leaves (18 d after germination in our conditions) (Boyes *et al.*, 2001). The first true leaves were infiltrated with *Agrobacterium tumefaciens* strain C58C1 carrying CaMV strain CM1841. Plants were scored for symptoms and photographed at 21 days post-infection (dpi). Symptoms were classified into categories (0–5) corresponding to no visible symptoms (0), mild vein clearing (1), leaf bending (2), rosette distortion (3), rosette shrinking (4), and early senescence with necrotic lesions (5), and were determined for each accession. Failed infections were removed from pots before taking aboveground fresh weights for individual plants. All infected plants (*n*=3–6) of one accession were pooled for titer measurements and ground to a fine powder in liquid nitrogen. Infections with the RNA viruses were performed using clones described in Ling *et al*. (2013) for turnip rosette virus (TRoV; family *Solemoviridae*) and Garcia-Ruiz *et al*. (2010) for turnip mosaic virus (TuMV; family *Potyviridae*).

### Virus quantification and gene expression analysis

For CaMV DNA quantification, 100 mg pulverized frozen leaf material was resuspended in 300 µl 100 mM Tris buffer (pH 7.5), supplemented with 2% SDS, and treated with Proteinase K. Total DNA was precipitated with isopropanol 1:1 (v:v). RNA extraction from rosette tissue was performed with a Qiagen RNeasy kit and on-column DNase I digestion according to the manufacturer’s protocol. Approximately 500 ng of total RNA was used for first-strand cDNA synthesis with a Maxima First Strand cDNA Synthesis Kit (Thermo Fisher Scientific,4 Waltham, MA, USA). Quantitative real-time PCR (qRT–PCR) analysis of DNA and cDNA was performed with Maxima SYBR Green/Fluorescein qRT–PCR Master Mix (Thermo Fisher Scientific) using the CFX Connect Real-Time PCR detection system (Bio-Rad, Hercules, CA, USA) with specific primers (Supplementary Table S3). Viral DNA was normalized to genomic *ACTIN7* (AT5G09810) for all accessions and *18S* ribosomal DNA for T-DNA lines. Viral transcripts and ABA-responsive transcripts were normalized to *PP2a* (*AT1G69960*).

### Genome-wide association mapping

Genome-wide association (GWA) mapping was performed on 100 accessions using an online portal provided by the Gregor Mendel Institute, Austria (https://gwas.gmi.oeaw.ac.at) (Seren *et al.*, 2012) against the Imputed Fullsequence Dataset (Cao *et al.*, 2011; Gan *et al.*, 2011; Long *et al.*, 2013) with an accelerated mixed model (AMM) (Seren *et al.*, 2012). The AMM is based on EMMAX (Kang *et al.*, 2010) and P3D (Zhang *et al.*, 2010), correcting for population structure and accounting for genetic relatedness as a random effect, but differs in the re-estimation of *P*-values for the 100 most significant single nucleotide polymorphisms (SNPs) through exact inference (Kang *et al.*, 2008). For details, refer to Seren *et al*. (2012). Analysis was performed with untransformed data. For this report, SNPs were considered when they withstood a 5% false discovery rate by Benjamini–Hochberg–Yekultieli thresholding (Benjamini and Hochberg, 1995) and a minor-allele count of ≥5. Fifteen T-DNA lines were chosen for the highest-scoring SNPs that fell into gene bodies, caused missense mutations, and had available T-DNA insertions in NASC.

### Chemical treatments

For chemical treatments, ABA (Sigma-Aldrich, A1049) and nordihydroguaiaretic acid (NDGA) (Merck Chemicals and Life Science, 74540) were prepared in 99% ethanol for stock solutions. Seedlings (17 d old) were sprayed with dilutions of these solutions 24 h before infection. The treatment was repeated once a week at the same time until harvest. The last application was performed 24 h before harvest.

### Broad-sense heritability calculation

The estimation of broad-sense heritability (h2b) was calculated as the percentage of the total variance accounted by genetic (accession) differences (h2b = σ2 G/σ2 P, where σ2 G is the genetic variance component of σ2 P total phenotypic variance). σ2 P and σ2 G were derived by variance components analysis using separated univariate analyses (Shukla *et al.*, 2018).

### Transcriptome analysis

Transcriptome data were generated by Chesnais *et al*. (2022). For the re-analysis of the bulk RNA-seq data, raw data were downloaded from BioProject number PRJEB49403 from the European Nucleotide Archive (https://www.ebi.ac.uk/ena/browser/view/PRJEB49403). Analysis was done on three replicates of mock- and CaMV-infected samples. In brief, downloaded reads were trimmed and checked with TrimGalore [version 0.5.0; https://github.com/FelixKrueger/TrimGalore, based on Cutadapt (Martin, 2011)] using the options -q 20 --fastq --stringency 1 --length 32 --paired. Afterwards, reads were mapped to the TAIR10 genome using Tophat2 (version 2.1.1; Kim *et al.*, 2013) with the parameters --library-type=fr-firststrand -g 1 -a 10 -i 40 -I 5000 -r 150, using the TAIR10 reference annotations for all annotated genes. Mapped output files were sorted and indexed using samtools (version 1.6; Li *et al.*, 2009). FeatureCounts from the subread package (version 2.0.1; Liao *et al.*, 2014) was used with the options -T 8 -p -t gene -O -s 2 against all genes in the TAIR10 genome to generate a counts table for subsequent analysis of differentially expressed genes using the R package Deseq2 (Love *et al.*, 2014).

### Bioinformatics

Plots were made with R 4.0.2, using the packages ‘ggplot2’ (Wickham, 2016), ‘tidyverse’ (Wickham *et al.*, 2019), ‘raincloudplot’ (Allen *et al.*, 2019), or base functions. All statistical calculations were performed in R with base functions. Test statistics can be found in Supplementary Table S4. Figure arrangements were finalized using AffinityDesigner 1.10. Latitude and longitude data, as well as SNP data and impact prediction, were taken from the https://1001genomes.org/ website and the POLYMORPH1001 tool (https://tools.1001genomes.org/polymorph/index.html) (Supplementary Table S1). Gene loci and descriptors were assembled through the PANTHERDB website version 16.0 using bedtools v2.30.0 ‘closest’ function.

## Results

### CaMV disease severity is highly variable in Arabidopsis

**Fig. 1.**
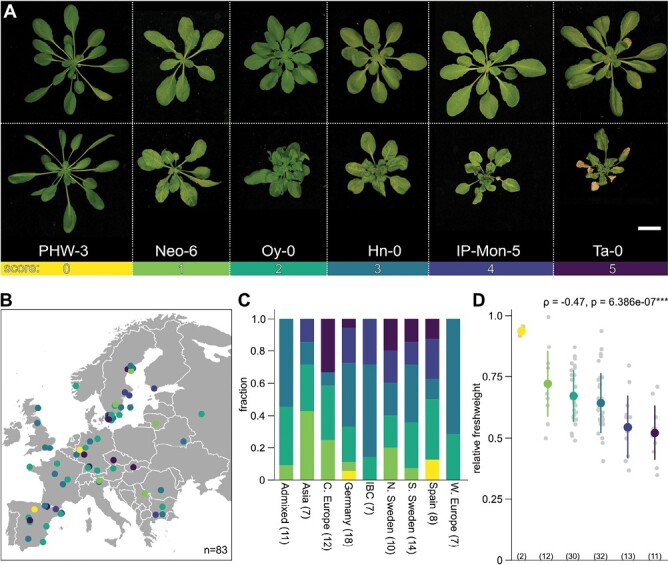
The broad spectrum of CaMV disease in Arabidopsis. (A) Representative images of the range of symptoms induced by CaMV infection at 21 dpi. Upper panel: mock-infected plants; lower panel: CM1841-infected plants. Accession identifiers are shown below. Colors correspond to symptom categories. Scale bar=2 cm. (B) Geographical distribution of 83 Arabidopsis accessions from Europe, representing 83% of the examined accessions. Dot colors indicate symptom categories. (C) Fraction of symptom categories divided by admixture groups. The number of accessions in each admixture group is indicated in brackets. IBC, Italy/Balkans/Caucasus; C, Central; N, North; S, South; W, Western. (D) Dot plot of relative fresh weights of accessions in the different symptom categories. Numbers in brackets indicate the number of accessions in each category. The colored circle and line represent the mean ±SD. Grey dots represent individual accessions. Correlation was calculated with the Spearman rank test.

CaMV occurs worldwide and infects Arabidopsis and other *Brassicaceae* in wild populations (Raybould *et al.*, 1999; Pagán *et al.*, 2010). In this study, we examined CaMV disease in 100 Arabidopsis accessions under controlled conditions. Accessions exhibited a broad range of symptoms that were scored at 21 dpi. We categorized symptoms from mild vein clearing (1) and leaf bending (2) through rosette distortion (3) and rosette shrinking (4) to early senescence with necrotic lesions (5) (Fig. 1A; Supplementary Fig. S1C). Only two accessions, PHW-3 and IP-Oja-0, did not develop any visible disease (score 0), while most accessions developed moderate symptoms (Supplementary Table S5). Of the 100 tested accessions, 83 were collected in Europe (Fig. 1B; see Supplementary Fig. S1A for a world map), but we could not find clustering of similar disease severities along either the longitudinal or the latitudinal gradient (Fig. 1B), and the main admixture groups (*n*>5) in our dataset did not reveal a pattern in symptom severity (Fig. 1C). Relative fresh weight after virus infection is a widely used proxy for disease severity, and it was strongly correlated with the visually determined disease categories in our dataset (Fig. 1D, Supplementary Table S5). Importantly, virus-induced fresh weight loss did not correlate with the total fresh weight of mock-inoculated plants, indicating that virus disease costs in these conditions are not dependent on differences in growth capacity between individual accessions (Supplementary Fig. S1B). We also tested a few accessions under different light conditions to evaluate the robustness of accession-specific symptomology and found that the range of symptoms was essentially reproduced (compare Fig. 1A and Supplementary Fig. S1C). These results reveal a large spectrum of CaMV-induced symptoms in Arabidopsis that appear to be largely independent of the global origin of the accession when grown in controlled conditions.

### Tolerance and resistance govern CaMV disease in Arabidopsis

**Fig. 2.**
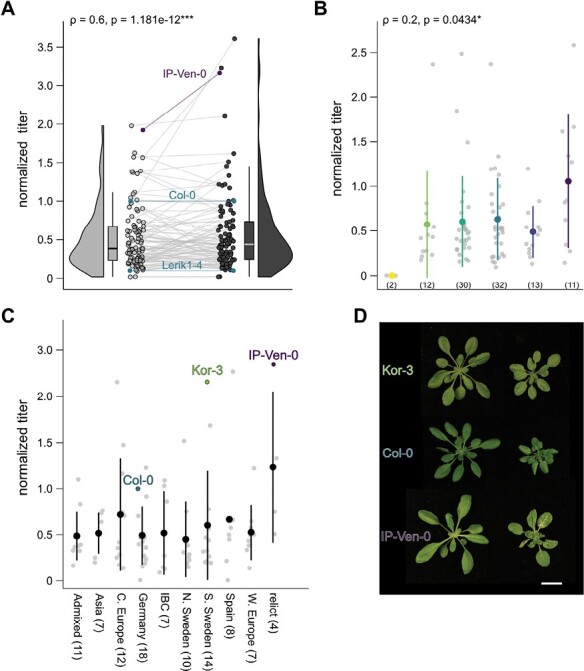
CaMV accumulation correlates only weakly with symptoms in Arabidopsis. (A) Raincloud plot of CaMV DNA accumulation in 100 Arabidopsis accessions at 21 dpi, in two independent replicates. The Spearman correlation coefficient (ρ) and *P*-value are given. (B) Dot plot of CaMV DNA accumulation in different symptom categories. The numbers in brackets indicate the number of accessions in each category. Colors correspond to symptom categories (see Fig. 1A). The colored circle and line represent the mean ±SD. Grey dots represent individual accessions. The Spearman correlation coefficient (ρ) and *P*-value are given. (C) Dot plot of CaMV DNA accumulation in admixture groups. Accessions depicted in (D) are highlighted. The number of accessions in each admixture group is indicated in brackets. The circles and lines represent the mean ±SD. Grey dots represent individual accessions. (D) Representative image of accessions Kor-3 and IP-Ven-0, with Col-0 for comparison. Both accessions accumulate twice as much CaMV DNA as Col-0 but fall on either side of Col-0 on the disease spectrum. Scale bar=2 cm.

To evaluate the relationship between virus accumulation and disease symptomology, we determined viral genomic DNA levels in parallel with the symptom scoring and fresh weight analysis presented in Fig. 1. CaMV DNA accumulation was measured from pools of infected plants from the two replicate experiments, with good reproducibility (Fig. 2A, Supplementary Table S6). We detected a 28-fold difference between the highest (IP-Ven-0) and the lowest (Lerik1-4) viral DNA measurement in symptomatic plants. Interestingly, we found only a weak correlation between viral titer and plant symptoms, and the four highest CaMV accumulators belonged to symptom groups 1, 2, 3 and 5, indicating that virus multiplication and virulence are largely uncoupled in the present setting (Fig. 2B). Likewise, several accessions from the severe symptom categories (4 and 5) accumulated low levels of virus, suggesting hypersensitivity. An equally poor but positive correlation between symptoms and viral accumulation has been previously described for the potyvirus TuMV (Rubio *et al.*, 2019), while no correlation was found for the cucumovirus cucumber mosaic virus (CMV) (Pagán *et al.*, 2007), altogether strengthening the idea that disease symptoms are frequently not a consequence of the amount of virus in a plant. We also could not detect differences in CaMV accumulation between the different admixture groups (Fig. 2C) other than a slightly higher value for relics, but the low number of accessions in this group might confound the effect. Again, the highest CaMV accumulators were scattered between the admixture groups. These data show that many Arabidopsis accessions vary in their tolerance of CaMV in a manner that is largely uncoupled from accumulation and, thus, that symptom development in individual accessions is far from a direct indicator of CaMV accumulation (Fig. 2D).

A recent study by Liu *et al*. (2022) examined the quantitative resistance of Arabidopsis to two distantly related strains of CMV; 41 (for CMV-Q) and 42 (Fny-CMV-Δ2b) accessions were shared between that study and the present work. Interestingly, while no correlation could be detected between CaMV and CMV accumulation in general, individual accessions, such as IP-Ven-0, accumulated high virus loads in both cases, and IP-Oja-0 showed full resistance to CaMV and accumulated very low levels of CMV (Supplementary Fig. S2). Another study on CMV virulence in Arabidopsis accessions from the Iberian peninsula also found that CMV infection in IP-Ven-0 drastically reduced seed production (by 96.5%), whereas IP-Oja-0 seed production was decreased by only 20% after infection (Montes *et al.*, 2021). The absence of a global correlation between CaMV and CMV accumulation across the accessions suggests that individual plant–virus interactions are commonly of high importance, but single accessions might still exhibit strong resistance or susceptibility to viruses generally, possibly as a consequence of physiological traits.

### Genome-wide association mapping identifies novel CaMV susceptibility factors

**Fig. 3.**
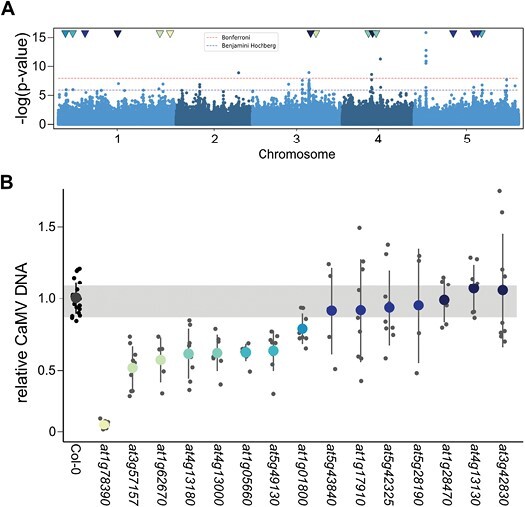
GWA mapping of CaMV accumulation and candidate screening. (A) Manhattan plot of GWA results for CaMV accumulation in 100 natural accessions of Arabidopsis. Blue shading indicates the five Arabidopsis chromosomes. The blue horizontal line indicates the significance threshold after Benjamini–Hochberg correction; the red line represents the more stringent Bonferroni multiple testing correction. (B) Relative CaMV DNA accumulation in T-DNA lines of GWA candidates (indicated by ATG number) at 21 dpi compared with wild-type Col-0 (*n*=4–22). T-DNA lines are listed in Supplementary Table S2. The colored circles and lines represent the mean ±SD. Grey dots represent individual accessions. The grey bar represents the standard deviation of Col-0.

We used the GWAPP tool (Seren *et al.*, 2012) to conduct a GWA mapping of symptoms, relative fresh weight, and relative CaMV accumulation in the 100 accessions. It is important to note that 100 accessions represents a small sample size for GWA mapping, which will result in limited resolution. Neither symptom category nor relative fresh weight data resulted in the identification of SNPs above the Benjamini–Hochberg threshold (Supplementary Fig. S3); however, several regions were associated with CaMV accumulation (Fig. 3A, Supplementary Table S7). Broad-sense heritability for CaMV DNA accumulation was 0.58, similar to previous observations in plant–virus systems (Shukla *et al.*, 2018; Monnot *et al.*, 2021). After thresholding, we found 140 genes within a 2 kb region of significant SNPs for CaMV titer (Supplementary Table S8), in accordance with the multifaceted process of viral replication. Most associated genes had no annotated function in ThaleMine (v.5.1.0-20221003). A protein class ontology search on PantherDB.org (v17.0) showed that the largest group of genes (16) by protein class ontology encodes metabolite interconversion enzymes (PC00264), eight of which are oxidoreductases (PC00176), followed by protein-modifying enzymes (eight; PC00260) and transcriptional regulators (six; PC00264). Since viral replication and accumulation could be influenced by as yet unknown mechanisms, we did not want to limit our downstream analysis, and randomly selected 15 SNPs above the threshold located in gene bodies that caused missense mutations (indicated by the colored arrowheads in Fig. 3A; Supplementary Table S2), for which we analysed CaMV accumulation in Col-0 based T-DNA insertion lines.

**Fig. 4.**
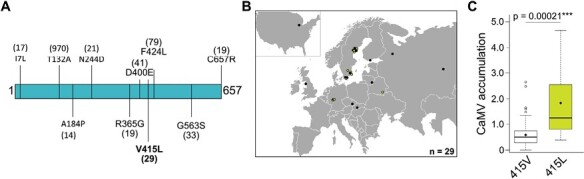
Allelic variation in NCED9 influences CaMV accumulation. (A) Graphic representation of NCED9 protein (657 amino acids) with amino acid substitutions due to SNPs present in more than 10 accessions annotated from the POLYMORPH 1001 browser. (B) Geographical distribution of 29 Arabidopsis accessions harboring NCED9-415L. Light green dots indicate accessions in our collection used for CaMV experiments. (C) CaMV DNA accumulation relative to Col-0 in NCED9-415V (*n*=95) and NCED9-415L (*n*=10) accessions. The *P*-value was calculated using a pairwise Wilcoxon rank rum test with continuity correction.

Intriguingly, of the 15 tested lines, eight showed a significant reduction in CaMV accumulation compared with Col-0 (Fig. 3B). It is noteworthy that none of the tested lines showed increased CaMV accumulation, suggesting that our GWA mapping mainly identified susceptibility factors. All lines developed symptoms similar to those in Col-0 at 21 dpi except for SALK_123975.34.85.x, which also had the most striking reduction of viral DNA (~5% of Col-0). This line harbors an insertion in the only exon of AT1G78390 (Lefebvre *et al.*, 2006). AT1G78390 encodes 9-*cis*-Epoxycarotenoid Dioxygenase 9 (NCED9), an enzyme involved in the biosynthesis of ABA. The identified SNP causes a missense mutation of valine-415 to leucine in the NCED9 coding sequence, with a predicted moderate effect (Fig. 4A). This particular polymorphism occurs in only 29 natural accessions, all of one are clustered in central and northern Europe and Russia (Fig. 4B; Supplementary Table S9). We expanded the virus accumulation analysis and additionally tested five further lines for accessions harboring this SNP (Supplementary Table S7). On average, accessions with NCED9-415L accumulated significantly more virus than those with NCED9-415V (Fig. 4C).

### NCED9 is essential for robust CaMV accumulation

**Fig. 5.**
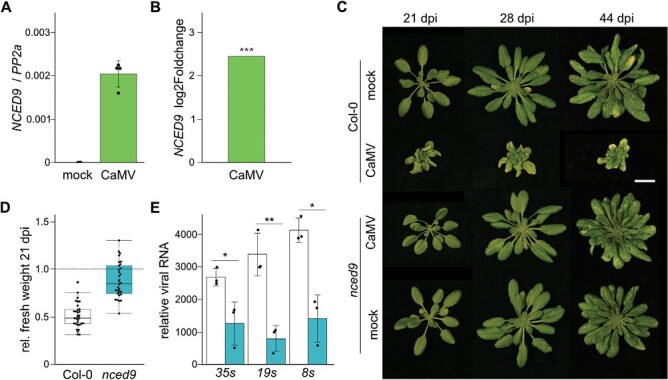
The *nced9* mutant is resistant to CaMV infection. (A) qRT–PCR of relative transcript accumulation of *NCED9* in mock- and CaMV-infected Col-0 plants at 21 dpi normalized to *PP2a* (*n*=4). (B) Log_2_ fold change of NCED9 in CaMV-infected compared with mock-infected plants in the transcriptome dataset of Chesnais *et al*. (2022). (C) Representative images of Col-0 and *nced9* plants at 21, 28, and 44 d after infection with CaMV strain CM184I. Scale bar=2 cm. (D) Relative fresh weight of infected Col-0 and *nced9* plants at 21 dpi. The black line indicates the mean fresh weight of mock-infected plants (normalized to a value of 1). (E) qRT–PCR of relative transcript accumulation of viral RNAs in Col-0 (white bars) and *nced9* (teal bars) plants at 21 dpi, normalized to *PP2a* (*n*=3).

NCED9 is best examined for its role during seed development and germination (Tan *et al*., 2003; Lefebvre *et al.*, 2006). We found that CaMV infection induced *NCED9* expression in rosette tissue compared with healthy plants, albeit still to low levels (Fig. 5A). We used an independent publicly available transcriptome set of Arabidopsis infected with the same CaMV strain, CM184I, from 21 d after aphid inoculation (Chesnais *et al.*, 2022) and could also find increased levels of *NCED9* transcript in response to CaMV (Fig. 5B). The *nced9* T-DNA line developed no symptoms except for a mild vein clearing phenotype in older leaves over an infection time of 44 d (Fig. 5C) and displayed no fresh weight loss compared with uninfected control plants when challenged with CaMV (Fig. 5D). This resistance phenotype was persistent also under long-day light regimes (Supplementary Fig. S4A). After backcrossing *nced9* into Col-0, we used symptom development to test whether homozygous *nced9* alleles are needed for CaMV resistance. Close to 90% of Col-0 plants developed symptoms upon infection, whereas 0% of homozygous *nced9* plants did. Three independent segregating F_2_ populations developed Col-0-like symptoms with a frequency of 69–72%, indicating that a homozygous line of *nced9* is required for CaMV resistance (Supplementary Fig. S4B). Plant resistance to viruses can be specific to the virus species and sometimes even the viral strain (Takahashi *et al.*, 2002). The *nced9* mutant is resistant to two strains of CaMV, the milder CM184I and the more virulent Cabb B-JI strain (Supplementary Fig. 4C), but is susceptible to infection with TuMV and TRoV (Supplementary Fig. S4D). Thus, NCED9 appears to be a CaMV-specific susceptibility factor. CaMV RNAs are very stable and can accumulate to high levels despite reduction in viral DNA (Hoffmann *et al.*, 2022). In *nced9,* all three major viral RNA species were reduced, albeit not as drastically as the viral DNA (Figs 3B, Fig. 5E).

### Exogenous ABA application enhances CaMV accumulation in Col-0

**Fig. 6.**
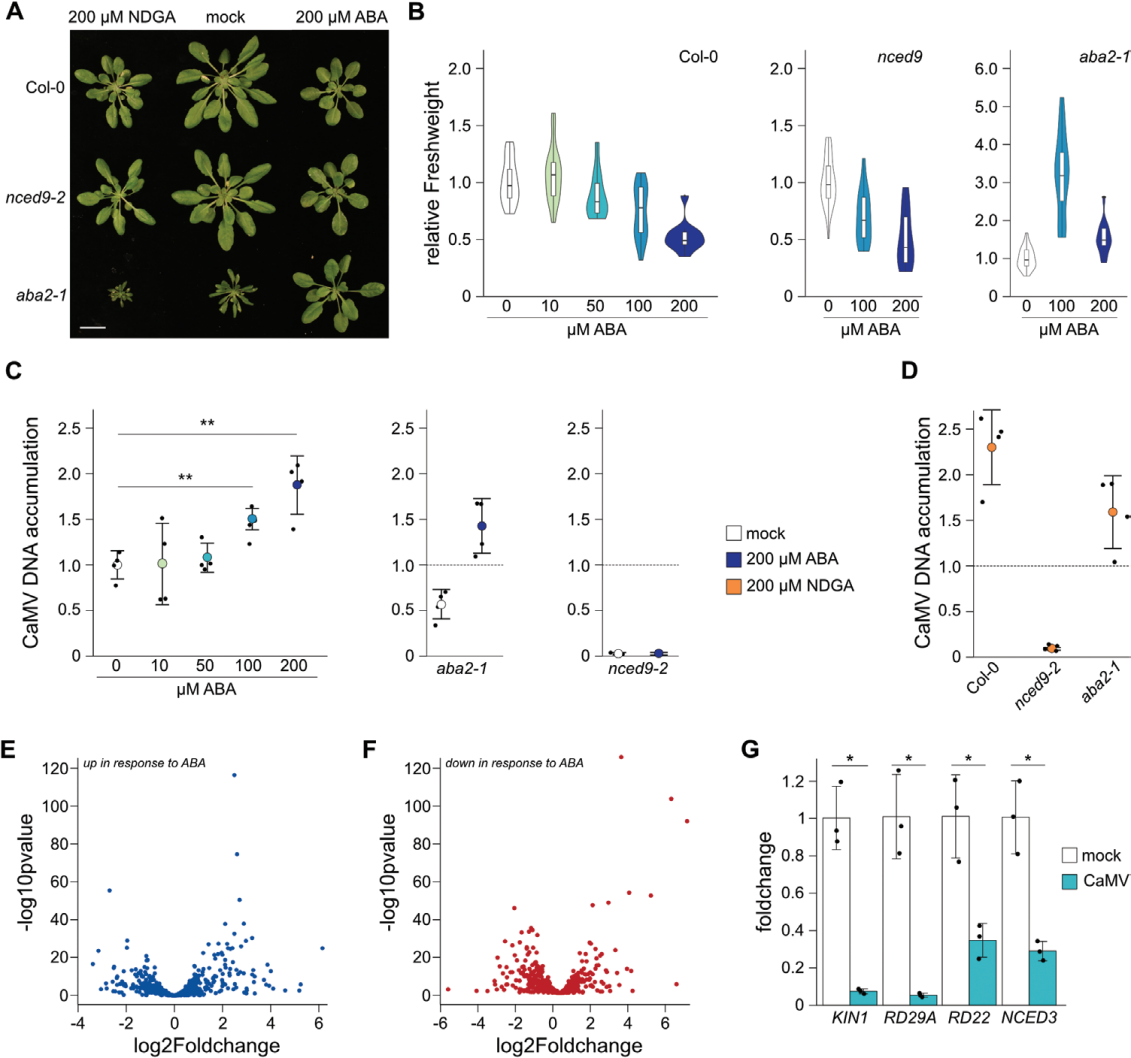
Exogenous application of ABA enhances CaMV accumulation in a dose-dependent manner. (A) Representative image of mock-inoculated plants after three treatments with either ABA or NDGA. Scale bar=2 cm. (B) Violin plot of the relative fresh weight of mock-inoculated Col-0 (left panel), *nced9* (middle panel), and *aba2* (right panel) plants after three treatments with the indicated concentrations of ABA. (C) Relative CaMV DNA accumulation at 21 dpi in Col-0 (left panel), *aba2* (middle panel), and *nced9* (right panel) after three treatments with the indicated ABA concentrations (*n*=4). (D) Relative CaMV DNA accumulation at 21 dpi in the indicated genotypes after three treatments with 200 µM NDGA (*n*=4). (E) Log_2_ fold change of ABA-responsive genes (‘up-regulated after ABA treatment’, *n*=651; Hoth *et al.*, 2002) in CaMV-infected compared with mock-infected plants in the transcriptome dataset of Chesnais *et al*. (2022). (F) Log*2* fold change of ABA-responsive genes (‘down-regulated after ABA treatment’, *n*=680; Hoth *et al.*, 2002) in CaMV-infected compared with mock-infected plants in the transcriptome dataset of Chesnais *et al*. (2022). (G) Relative transcript accumulation of ABA-responsive genes (from the category ‘up’) in Col-0 plants at 21 d after mock or CaMV infection, normalized to *PP2a* (*n*=3).

ABA is generated through the cleavage of C_40_ carotenoids by several enzymatic reactions, originating in the chloroplast and ending in the cytoplasm. The multigene NCED family encodes enzymes that cleave *cis*-isomers of violaxanthin and neoxanthin to xanthoxin, the last precursor of ABA generated in chloroplasts (Nambara and Marion-Poll, 2005). The established role of NCED9 in ABA biosynthesis prompted us to investigate the involvement of ABA during CaMV infection. ABA plays multifaceted roles during plant–pathogen interactions, and exogenous ABA application was found to either increase or reduce pathogen load *in planta* (Alazem *et al.*, 2014). We treated seedlings with ABA 24 h before infection with CaMV and then once a week throughout the 3-week infection time course, with the last treatment 24 h before harvesting of the whole rosette. Application of exogenous ABA by spraying reduced Col-0 rosette growth in a concentration-dependent manner (Fig. 6A, B). The *nced9* plants behaved comparably to Col-0, showing that the line has not lost its sensitivity to ABA (Fig. 6A, B). The well-described *aba*2 mutant accumulates ~20–25% of wild-type ABA levels during undisturbed growth (González-Guzmán *et al.*, 2002), has a severely impaired growth phenotype, and is prone to wilting (Fig. 6A). The *aba2* growth phenotype was fully rescued by exogenous ABA spraying, suggesting that this treatment was applied successfully (Fig. 6B). CaMV accumulation in Col-0 was not affected after spraying with low concentrations of ABA (10 µM or 50 µM), but 100 µM and, more strongly, 200 µM increased the viral DNA content (Fig. 6C). Likewise, while virus levels were reduced in non-treated *aba2-1* plants, virus load significantly increased upon spraying with 200 µM ABA (Fig. 6C). Intriguingly, exogenous ABA application had no effect on CaMV accumulation in the *nced9* background, seemingly uncoupling the function of *NCED9* in CaMV infection from bulk ABA synthesis (Fig. 6C).

The phenolic antioxidant NDGA is a commonly used inhibitor of lipoxygenases (NCEDs) and as such is an inhibitor of ABA synthesis (Creelman *et al.*, 1992; Han *et al.*, 2004). NDGA has been used previously in plant–virus studies and either made the plants more susceptible to the virus (He *et al.*, 2021) or reduced viral load *in planta* (Alazem *et al.*, 2014). We observed that NDGA treatment decreased plant growth in uninfected plants (Fig. 6A), but also that NDGA treatment increased CaMV DNA accumulation in Col-0 and *aba2-1*, whereas it had no effect on virus accumulation in *nced9* (Fig. 6D). These results suggest that disturbance of ABA homeostasis, rather than ABA levels, might aid virus accumulation. We used CaMV transcriptome data (Chesnais *et al.*, 2022) to visualize the effect of CaMV infection on ABA-responsive genes in 4-week-old rosettes (Hoth *et al.*, 2002). CaMV infection altered the expression of positively and negatively ABA-regulated genes drastically and in a non-specific manner, indicating a disturbance in ABA signaling pathways (Fig. 6E, F; Supplementary Table S10). To validate that these changes hold true in our experimental conditions, we chose four ABA-responsive genes that are down-regulated during CaMV infection according to the transcriptomics data and tested their expression with qRT–PCR, confirming their strong transcriptional repression during CaMV infection (Fig. 6G). Taken together, our data suggest that CaMV infection benefits from the disturbance of ABA homeostasis, probably through the misregulation of ABA-dependent pathways that ultimately helps viral accumulation. However, the function of NCED9 for CaMV as part of these ABA-related mechanisms remains to be determined.

## Discussion

**Fig. 7.**
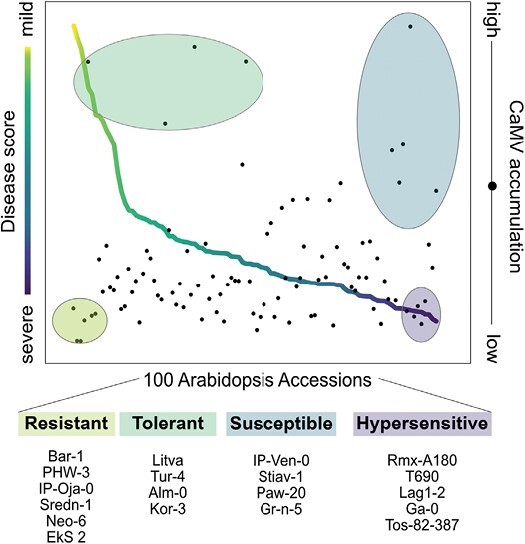
Tolerance and resistance shape CaMV disease in *Arabidopsis thaliana*. Line plot of disease score (relative fresh weight/ symptom category) color coded by symptom category, overlaid with a scatterplot of CaMV accumulation within the same accession. Identified groups are circled and color coded for their response. Accessions within the circles are listed below the graph, named by their accession identifier.

Plants can exhibit amazing plasticity in response to pathogens and the Arabidopsis/CaMV pathosystem is no exception. Arabidopsis is a natural host of CaMV, yet it remains speculative whether Arabidopsis evolved under CaMV pressure, as has been proposed for other viruses that naturally infect Arabidopsis (Montes *et al.*, 2019). In our conditions, Arabidopsis exhibited a wide spectrum of responses to CaMV that ranged from no symptoms and no viral accumulation to full susceptibility with strong symptoms and high viral accumulation. Notably, we found tolerant and hypersensitive accessions as well, once again exemplifying that symptom severity and virus accumulation are largely uncoupled between host genotypes and that both resistance and tolerance mechanisms shape plant–virus interactions (Fig. 7) (Pagán *et al.*, 2007; Rubio *et al.*, 2019; Bergès *et al.*, 2021). The defiance of pathogen-load/symptom connections (‘tolerance’) has been reported in other infection systems, including bacteria and fungi (Chen *et al.*, 2004; Gambetta *et al.*, 2007), although examples of clear resistance trajectories also exist, for example, for *Pseudomonas syringae* on Arabidopsis, which shows a strong positive correlation between symptom severity and bacterial density (Kover and Schaal, 2002). CaMV causes moderate symptoms in most accessions, a trend also seen with TuMV in 1050 Arabidopsis accessions (Butković *et al*., 2022, Preprint) and in line with the theory that viruses evolve for intermediate severity to balance viral replication and host survival (Anderson and May, 1982; Torres-Barceló *et al.*, 2010).

In our study, only two accessions, PHW-3 and IP-Oja-0, were fully resistant to CaMV infection (Fig. 2B). Previously, four more accessions (En-2, Wil-2, Sv-0, and Tsu-0) had been reported to be CaMV resistant (Leisner and Howell, 1992). Importantly, resistance phenotypes are often virus-strain specific. In accordance, the En-2 resistance locus (CAR1) on chromosome 1 (Callaway *et al.*, 1996) is broken by the P1 protein of CaMV strain NY8153 (Adhab *et al.*, 2018), while resistance in Tsu-0 is broken by P6, pointing towards individual resistance mechanisms among the accessions (Hapiak *et al.*, 2008). The identification of additional CaMV-resistant accessions will enable the identification of underlying resistance loci and help in the discovery of pathways implicated in plant–virus interactions.

Virus disease in plants is affected by the environment, as well as the genotype of the virus and host, forming a ‘disease triangle’. Manipulation of any factor in this triangle will affect the outcome of virus infection (Hily *et al.*, 2016). By controlling for environmental factors in standardized laboratory conditions, as well as for virus genotype by directed infiltration, we could elucidate the effect of host genotype on CaMV infection in Arabidopsis through GWA mapping. Previous GWA mappings in Arabidopsis/virus systems have identified resistance loci for the potyvirus TuMV, including the well-studied *RESTRICTED TEV MOVEMENT 3* (*RTM3*) gene (Cosson *et al.*, 2010; Pagny *et al.*, 2012; Rubio *et al.*, 2019), and novel regulators of RNA silencing during CMV infection (Liu *et al.*, 2022). Our GWA mapping identified numerous SNPs associated with differences in CaMV accumulation, in agreement with the diverse challenges that virus infections impose on host cells (Fig. 3A). Importantly, no resistance gene is known for Caulimoviruses, except the *CAR1* locus in the Arabidopsis En-2 accession, which has not been further mapped (Adhab *et al.*, 2018). Nonetheless, several genes involved in various cellular homeostatic processes have been identified through genetic studies that influence CaMV accumulation (Love *et al.*, 2005; Schepetilnikov *et al.*, 2011; Hafren *et al.*, 2017; Shukla *et al*., 2021, Preprint; Hoffmann *et al.*, 2022). Eight out of the 15 T-DNA insertion lines that we tested displayed reduced CaMV accumulation in the Col-0 background compared with wild-type Col-0 (Fig. 3B), a surprisingly high number considering that SNPs identified via GWA are frequently effective only in their natural genetic background (Corwin *et al.*, 2016; Gallois *et al.*, 2018). Notably, none of these eight genes had previously been associated with CaMV disease, underscoring the potential of GWAS to uncover hidden CaMV disease genes. All identified SNPs appear to be susceptibility factors for CaMV, as their deletion negatively affects virus accumulation. This could point to either the importance of recessive resistance to CaMV, or more efficient identification of susceptibility factors in our GWAS. Even though the identified SNP for *NCED9* has a low allele frequency and was not among the highest-scoring ones, the *nced9* mutant had by far the greatest effect and is, to our knowledge, the most CaMV-resistant Arabidopsis T-DNA insertion mutant identified so far. The same T-DNA line has been commonly used and well described for ABA experiments during seed germination, where *NCED9*, together with *NCED6*, is the main biosynthesis gene (Tan *et al*., 2003; Lefebvre *et al.*, 2006). To our surprise, CaMV infection in *nced9* could not be rescued by application of exogenous ABA by spraying, unlike the *aba2-1* mutant (Fig. 6C). Two other viruses were able to systemically spread through *nced9* and cause wild-type-like symptoms, indicating that the resistance is specific for CaMV (Supplementary Fig. S4D). While we could not determine which function of NCED9 is essential for CaMV infection, the drastic defect in *nced9* mutant warrants more attention.

Interestingly, even though the virus accumulation defect in *nced9* could not be alleviated by exogenous ABA application during CaMV infection, ABA hormone levels had an impact on CaMV accumulation. Plant hormones are an integral part of signaling mechanisms in response to biotic and abiotic environmental stimuli (Verma *et al.*, 2016). The level and inducibility of hormonal responses exhibits a large range between Arabidopsis accessions, as identified for the major stress hormones salicylic acid (Bruessow *et al.*, 2021) and ABA (Kalladan *et al.*, 2017). Upon pathogen attack, ABA mediates the closure of stomata and deposition of callose at the plasmodesmata to slow the spread of the pathogen (Ton *et al.*, 2009). While callose deposition could reduce the plasmodesmal trafficking of CaMV, as observed for many other viruses (Iglesias and Meins, 2000; Li *et al.*, 2012; Zavaliev *et al.*, 2013), this is unlikely, as spraying with ABA increased systemic CaMV accumulation. Additionally, ABA antagonizes the salicylic acid-mediated systemic acquired resistance (SAR), which could make it a general target of pathogens to subvert the antimicrobial SAR (Yasuda *et al.*, 2008), and could be used by CaMV to escape SAR (Love *et al.*, 2005). However, for virus infections including CaMV, the role of ABA appears to be complex. Increased ABA content was measured in *Nicotiana tabacum* after TMV infection (Whenham *et al.*, 1986), in rice after rice stripe virus infection (Cui *et al.*, 2021), and with CMV (Alazem *et al.*, 2014) but not plum pox virus (PPV) infection in Arabidopsis (Pasin *et al.*, 2020). Further treatment with ABA increased plant resistance to tobacco mosaic virus (Chen *et al.*, 2013), PPV (Pasin *et al.*, 2020), Chinese wheat mosaic virus (He *et al.*, 2021), and bamboo mosaic virus (BaMV) in Arabidopsis (Alazem *et al.*, 2014), and reduced the lesion size in local infections of tobacco necrosis virus (TNV) (Iriti and Faoro, 2008). CaMV, on the other hand, accumulated to higher levels upon treatment of plants with ABA, in line with a reduction in the *aba2* mutant that was furthermore rescued by ABA application. However, the strong increase in CaMV accumulation upon treatment with NDGA, an inhibitor of the ABA biosynthetic NCED family, is difficult to understand, but notably, ABA and NDGA also acted similarly in that both reduced BaMV accumulation in Arabidopsis (Alazem *et al.*, 2014). Thus, our data suggest that CaMV benefits from disruption of ABA homeostasis; indeed, we also found that ABA-responsive genes are widely affected by CaMV and highly deregulated when compared with ABA treatment (Fig. 6E–G) (Hoth *et al.*, 2002). This could be at least partially attributable to the CaMV P6 protein interacting with and repressing the function of histone deacetylase H2DC, a regulator of ABA-mediated gene expression (Sridha and Wu, 2006; Li *et al.*, 2021).

Taken together, our findings demonstrate that GWA is a powerful tool to identify novel players in DNA virus disease. The large plasticity of Arabidopsis towards CaMV and the independent resistant lines indicate independently evolved resistance mechanisms that should be explored further. We found evidence that resistance as well as tolerance mechanisms play a role during CaMV infection. Finally, ABA was identified as a novel inducer of CaMV accumulation and CaMV infection drastically misregulates ABA-responsive genes.

## Supplementary data

The following supplementary data are available at *JXB* online.

Fig. S1. Additional information on Arabidopsis accessions.

Fig. S2. Correlations between CaMV and CMV data.

Fig. S3. Manhattan plots of GWA mapping for symptoms and relative fresh weight.

Fig. S4. Phenotypes of *nced9* and Col-0 plants in different growth conditions.

Table S1. Arabidopsis accessions used in this study.

Table S2. T-DNA lines used in this study with primers used for their confirmation.

Table S3. Primers used in this study.

Table S4. Statistics used in this study.

Table S5. Symptom and relative fresh weight data by accessions.

Table S6. Relative CaMV accumulation in two replicates by accessions.

Table S7. SNPs above GWA score 5 and MAC ≥5.

Table S8. Genetic elements in a 2 kb window of SNPs.

Table S9. Arabidopsis accessions harboring NCED9 415L.

Table S10. ABA-responsive genes and their expression during CaMV infection.

erad204_suppl_Supplementary_Figures_S1-S4

erad204_suppl_Supplementary_Tables_S1-S10

## Data Availability

All data supporting the findings of this study are available within the paper and within its supplementary data published online.
