# Supplementary material for: Cauliflower mosaic virus disease spectrum uncovers novel susceptibility factor NCED9 in Arabidopsis thaliana
Source: J Exp Bot. 2023 May 30;74(15):4751–64. doi: 10.1093/jxb/erad204 (PMC10433934; doi:10.1093/jxb/erad204)
Supplement: erad204_suppl_Supplementary_Figures_S1-S4 [file erad204_suppl_supplementary_figures_s1-s4.pdf]

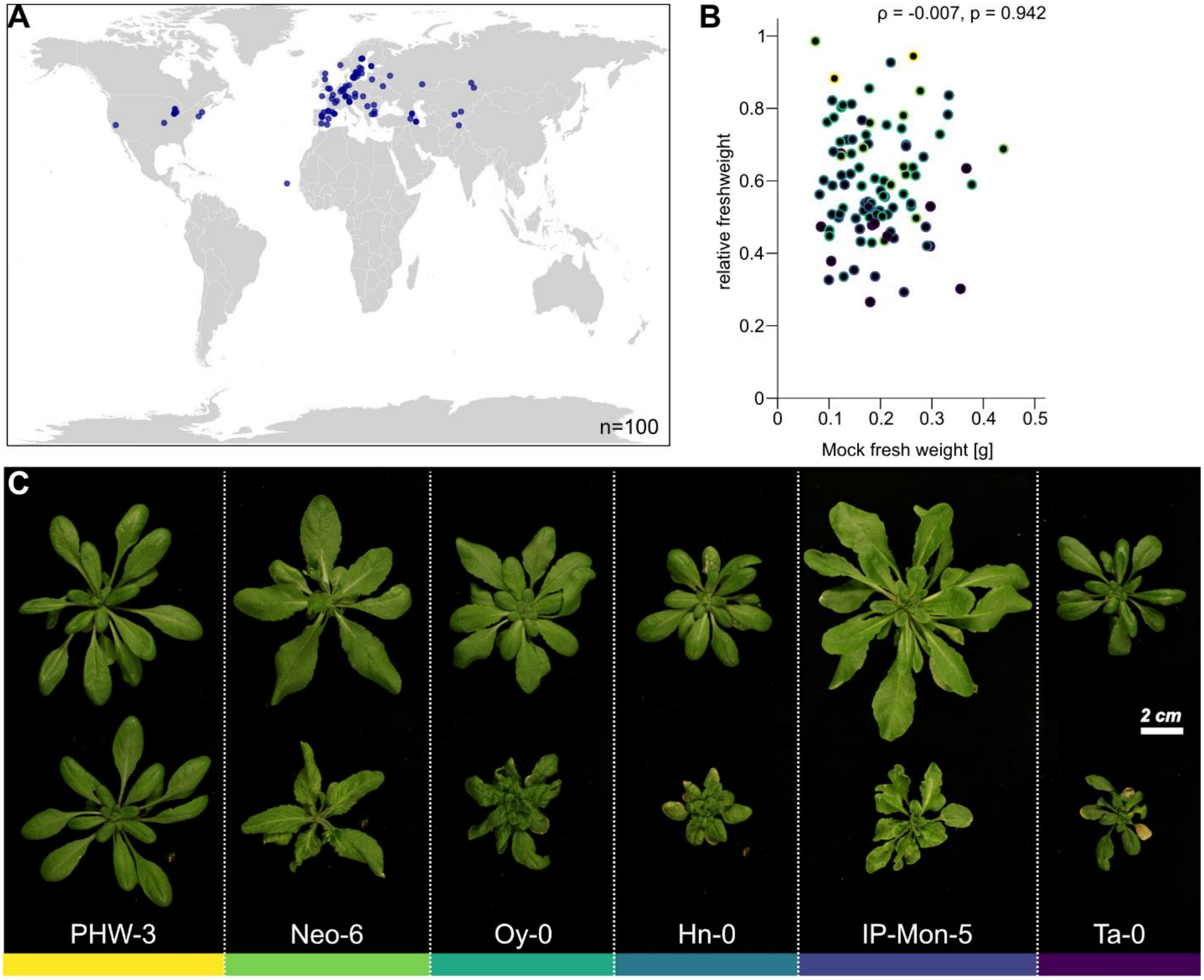

Supplementary Figure S1: Distribution of Arabidopsis accessions
(A) Worldwide geographical distribution of all Arabidopsis accessions used in this study.
(B) Correlation of relative FW in CaMV infected plants with the total fresh weight of mock plants.
(C) Representative images of symptom range induced by CaMV infection 21 dpi in plants grown in long day conditions. Neo-6 started transition to flowering. Upper panel: mock infected plants, lower panel: CM1841 infected plants. Accession identifier is written below. Colours correspond to symptom categories. Scale bar = 2 cm

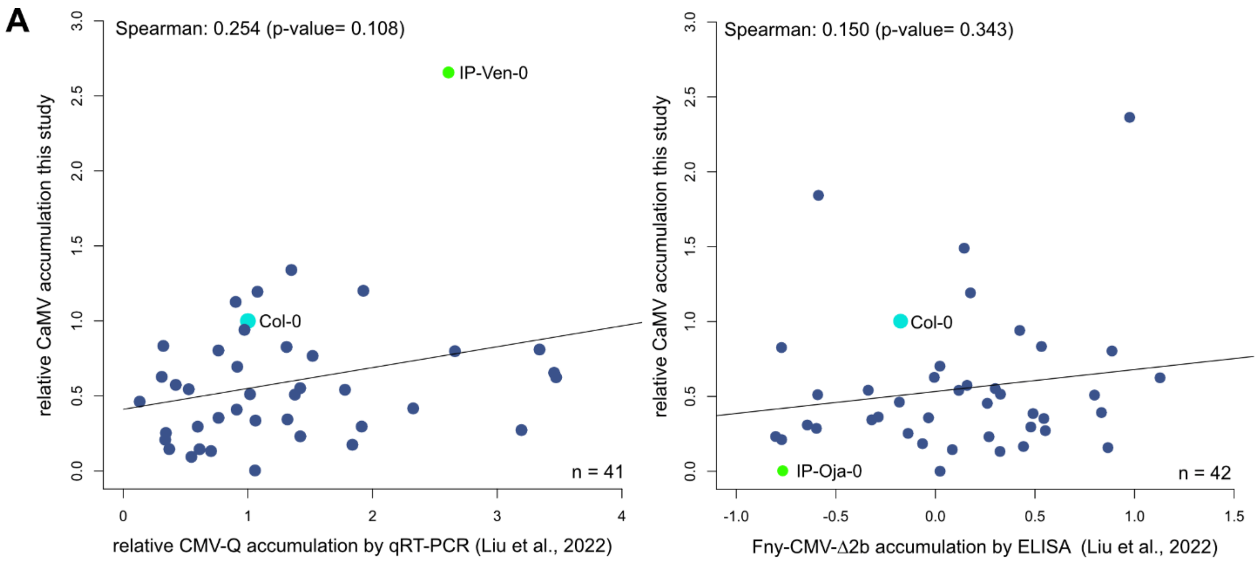

Supplementary Figure S2: Correlation between CaMV and CMV accumulation
(A) Scatterplots of virus accumulation shared between this study and Liu et al., 2022 (n=41 for CMV-Q and n=42 for Fny-CMV-2b). Both studies normalized virus accumulation to Col-0 (light blue dot).

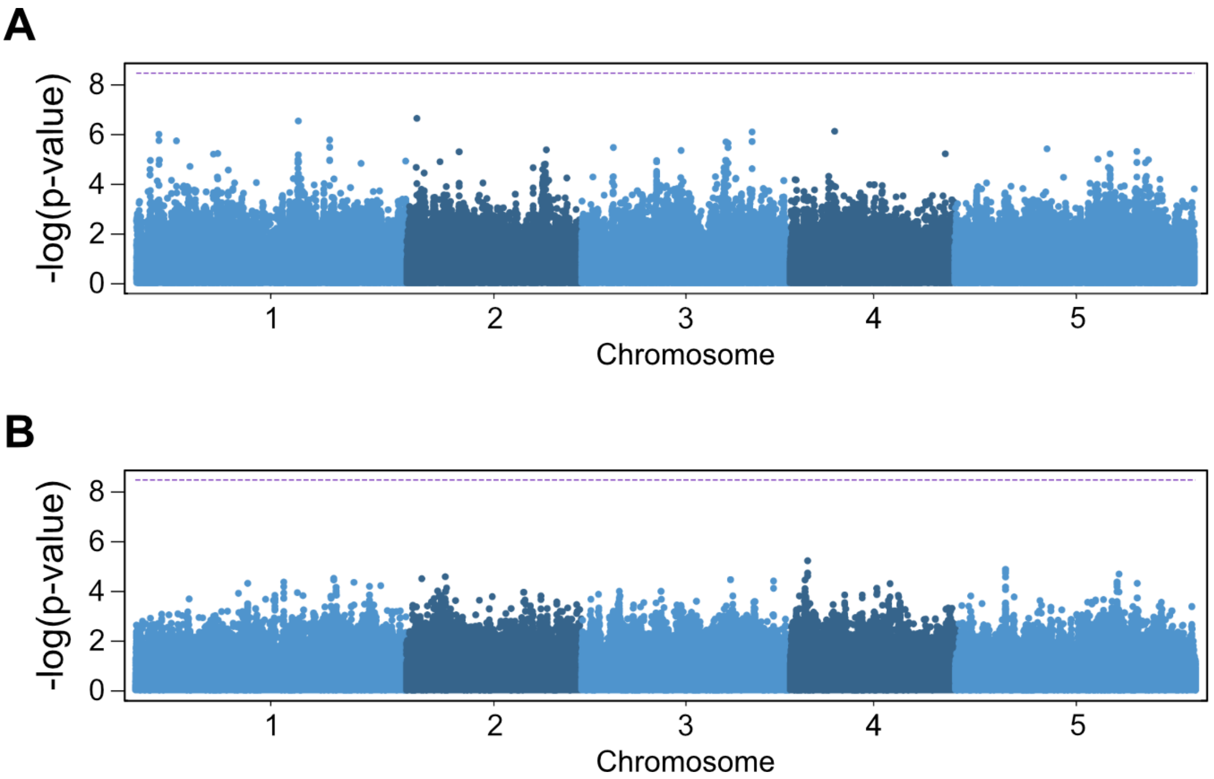

Supplementary Figure S3: GWA-mapping of symptom and relative fresh weight data
(A) Manhattan plot of GWA results for symptom categories in 100 natural accessions. **(B**) Manhattan plot of GWA results for relative fresh weight of virus infected plants in 100 natural accessions. Blue shading corresponds to the five Arabidopsis chromosomes. Blue lines indicate significance threshold after Benjamini-Hochberg correction, red line represents the more stringent Bonferroni multiple testing correction.

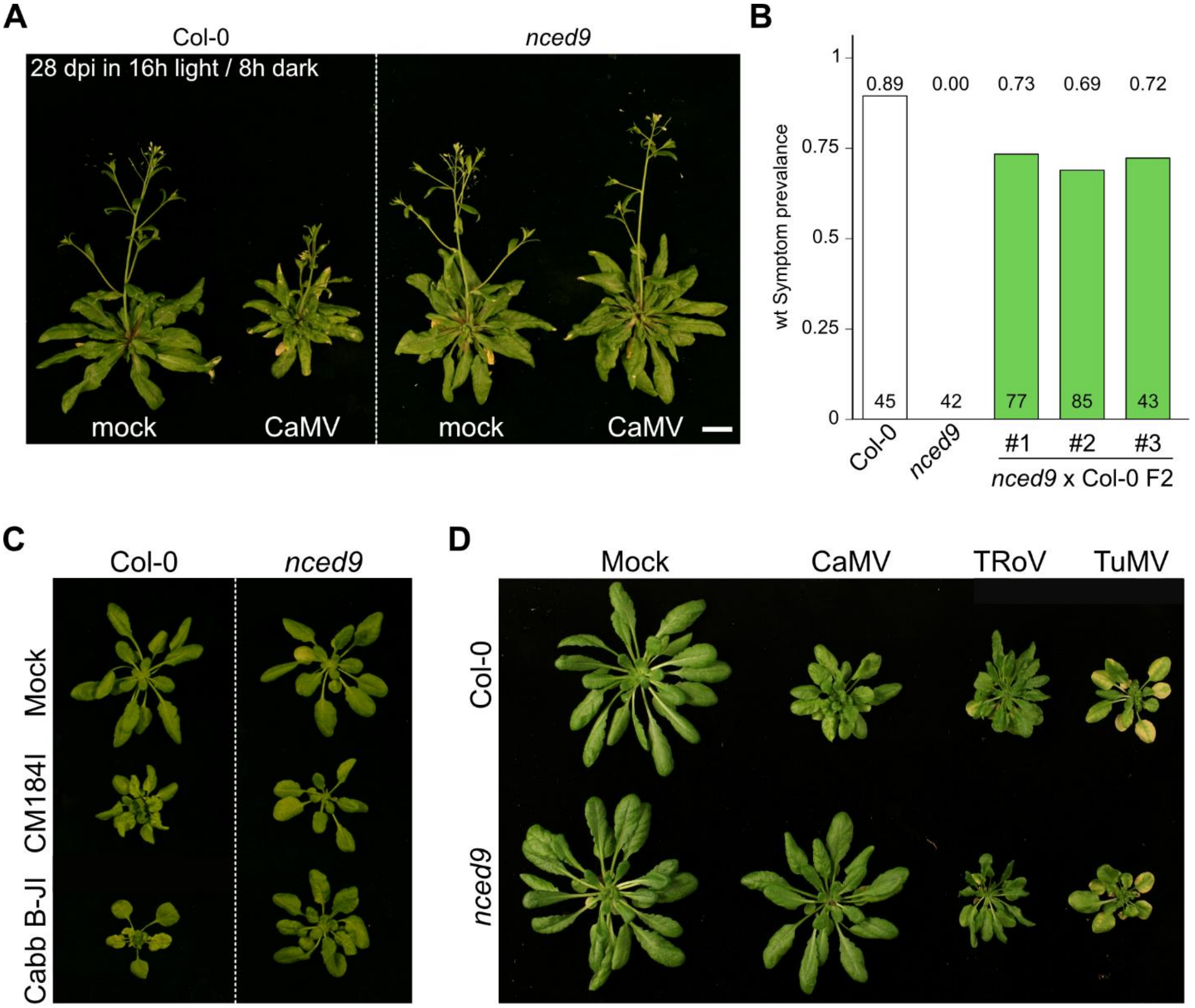

Supplementary Figure S4: Phenotype of *nced9* compared to Col-0 in different conditions

(A) Representative image of Col-0 and *nced9* plants grown in long day conditions at 28 dpi. Scale bar = 2 cm
(B) Prevalence of Wildtype-like symptoms in a segregating population of *nced9* x Col-0 crosses.
(C) Representative image of Col-0 and *nced9* plants infected with two strains of CaMV at 21 dpi.
(D) Representative image of Col-0 and *nced9* plants infected with three viruses at 21 dpi.
